# Physiology and pathology of the C3 amplification cycle: A retrospective

**DOI:** 10.1111/imr.13165

**Published:** 2022-11-21

**Authors:** Keith Peters

**Affiliations:** ^1^ The Francis Crick Institute London UK

**Keywords:** alternative pathway, amplification loop, complement, nephritic factor, tickover

## Abstract

The C3 “Tickover” hypothesis, a mechanism whereby the host maintains constant surveillance of potential invading pathogens, targeting them for elimination through amplified C3b generation and C3‐dependent effector mechanisms, was proposed by the late Professor Peter Lachmann in 1973. This unique insight came from a combined understanding of the complement system as it was then defined and the nature of the disease process in rare complement deficiencies and complement‐driven diseases. In this review, I give a personal perspective of how understanding of “Tickover” has developed in the subsequent 50 years, culminating in the introduction into the clinic of therapeutic agents designed to combat amplification‐driven disease.

## 1 INTRODUCTION

In the Spring of 1970, a few months after I had started as a Lecturer in Renal Medicine at the Royal Postgraduate Medical School (RPMS) at Hammersmith Hospital in London, I arranged to meet Peter Lachmann, then in Cambridge, to set up a program studying the metabolism of complement proteins in patients with immunological disease. I was fortunate that he was enthusiastic about the idea but doubly so because the following year he accepted the Foundation Chair of Immunology at the RPMS, where we established a close collaboration and a lifetime friendship. Peter had worked with Henry Kunkel at the Rockefeller and was imbued with Henry's philosophy of combining information gleaned from patients with molecular analysis in the laboratory, a perfect fit for the RPMS, an institution which attached the highest priority to clinical research. Peter spent 1971–6 at the Hammersmith before returning to Cambridge, but more than half a century later related research is flourishing at the Hammersmith, driven first by his graduate student Mark Walport and subsequently by Marina Botto and Matthew Pickering‐I refer to their work later. I feel greatly privileged to be asked to contribute this review and I want to take the opportunity to illustrate the many clues to complement physiology, its role in disease, and ideas for therapy that arose from the detailed study (in the Kunkel tradition) of a small number of patients with exceptionally rare diseases. In the 1970s, complement was not even considered a mainstream aspect of immunology, and international meetings were easily accommodated in coastal resort hotels. The complement world was transformed with the discovery of its potential role in age‐related macular degeneration (AMD) in 2005. Peter had a long‐term belief in complement therapeutics based on his ideas of modulating the physiology of the C3 feedback cycle. These eventually materialized in his 80 s with the formation in 2014 of Gyroscope Therapeutics, a gene therapy company.

## 2 A CASE REPORT, A CHANCE MEETING, AND A GOOD IDEA

Lachmann introduced the concept that the complement system existed in a state of low‐grade activation, likening it to the tickover of a car engine ready to accelerate immediately if need arises. This made sense in relation to the recognition that complement, more specifically its alternative pathway (the pathway that was not dependent on adaptive immunity through the generation of antibody) was a major constituent of the innate immune system. The pathway provides an immediate defense against pathogens and toxins which cannot await the development of adaptive immunity, a biological system of clear evolutionary advantage.

Early studies of C3 metabolism showed that there was indeed a very high turnover of C3, which has a half‐life of around 3 days, with similar values for other components of the system.^1^ But such a system needs physiological control to prevent excessive and potentially harmful generation of inflammatory complement molecules. At a meeting in Bruges when Chester Alper presented the now famous Boston patient, TJ, who suffered from recurrent bacterial infection, abnormalities of complement function, and profound reduction of C3,^2^ Lachmann suggested TJ be tested for what was then termed KAF (conglutinogen activating factor, now known as Factor I). Lachmann went on to show that TJ was totally deficient in Factor I (FI), and was able to reproduce the same complement profile in vitro by immunochemical depletion of FI. He further showed that purified FI restored TJ's complement function in vitro and in vivo.^3^, ^4^ Many years later Lachmann et al. showed that an antibody which specifically reacted with C3b but not C3, inhibited binding of C3b to Factor B (FB), and prevented the complement activation induced by immunochemical FI depletion in vitro.^5^

The TJ experiments marked the beginning of elucidation of the molecular physiology of the C3 feedback loop, where complement tickover is the result of the continuous generation of the C3 convertase, C3bBb. A widely reported mechanism for this is the slow hydrolysis of C3 producing C3(H_2_O)^6^ which has C3b‐like characteristics and reacts with FB; but tiny quantities of a variety of proteolytic plasma enzymes such as plasmin or neutrophil elastase, or minimal activation of the classical or lectin pathways, would suffice. Factor D acts by converting C3b‐bound FB to Bb, and Properdin by stabilizing C3bBb. Amplification of the cycle occurs whenever C3 activation, brought about by any of the pathways, classical, alternative, or lectin pathways,^7^ exceeds its inactivation. It is therefore a mistake to equate the feedback cycle with the alternative pathway.^8^

One essential feature of the alternative pathway is that its activators accelerate the feedback loop by blocking its inhibition, as initially demonstrated by Fearon who showed that the archetypal alternative pathway activator, zymosan, did so by inhibition of surface recruitment of factor H (FH).^9^ The attachment of FH to cell surfaces provides an elegant mechanism to distinguish self from foreign surfaces such as those of bacteria^10^: Complement activation occurs on the surface of a pathogen but not on the host cells. Supportive evidence of the physiological existence of the tickover was provided by the elucidation of the molecular pathology of paroxysmal nocturnal hemoglobinuria (PNH). PNH cells are susceptible to complement‐mediated hemolysis because they lack the GPI anchored inhibitors CD59 and CD55 (Decay Accelerating Factor, DAF), a process that is dramatically arrested by the anti‐C5 antibody eculizumab.^11^ C3 turnover studies in PNH showed no difference from normal,^1^ consistent with the notion that normal tickover, in the absence of down‐regulation (by CD55), is sufficient to lyse the complement sensitive cells of PNH.

Inhibitory control of C3bBb is not only dependent upon plasma factors H and I, but also a variety of tissue factors including CD55 (mentioned above) and the complement receptors membrane co‐factor protein (MCP,CD46) and complement receptor 1 (CR1, CD35). CR1 is present in the circulation, principally on red blood cells, and in the kidney on glomerular podocytes.^12^ In vitro complement experiments are usually conducted on plasma or serum, in the absence of CRI, and therefore may not fully reflect events in vivo. The central streaming of flow of red blood cells in small blood vessels such as venules might also have pathophysiological implications by leaving the endothelium relatively unprotected by erythrocyte CR1.

## 3 ANOTHER CASE REPORT

In 1981, Thompson and Winterborn reported genetic deficiency of FH in an 8‐month‐old boy with atypical hemolytic uremic syndrome (aHUS).^13^ His plasma and serum complement profile closely resembled that of the FI deficiency of TJ, and subsequently it has been established that FH and related deficiencies of the amplification loop, including FI and MCP, and gain of function mutations in C3 and FB, provide the genetic basis of this disease.^14^, ^15^ In aHUS the occlusion of glomerular capillaries by platelets and fibrin leads to fragmentation of erythrocytes and severe impairment of glomerular filtration, and provides clear evidence of the links between the coagulation and complement systems.^16^ The recognition of the causal role complement deficiency and pathological complement activation plays in aHUS led to a major therapeutic advances in this field, initially through plasma exchange to provide deficient factors, then by the anti‐C5 monoclonal antibody eculizumab.^17^

## 4 NEPHRITIC FACTORS (NEF)

In 1969, a C3 splitting factor termed C3Nef was found in patients with a rare form of nephritis characterized by persistent low plasma C3 but normal levels of the early components of the classical pathway.^18^ Persistent hypocomplementemia was a feature of a subacute nephritis, histologically categorized as membranoproliferative (MPGN) or mesangiocapillary nephritis (MCGN).^19^ There was clear evidence of complement activation with C3 breakdown products detectable in fresh plasma.^18^, ^20^ The nature of Nef was initially uncertain but it was inseparable from IgG^21^, ^22^ and was eventually showed to be an autoantibody to a neo‐epitope on C3bBb, greatly prolonging C3bBb half‐life by inhibiting its breakdown by Factors H and I,^23^, ^24^, ^25^, ^26^ and leading to a plasma complement profile similar to that of patient TJ discussed above.

More detailed immunohistological and electron microscopic studies of renal pathology revealed distinct subgroups, namely those with a typical immune complex disease, with prominent deposits of immunoglobulin (Ig), MPGN, those without or only trace Ig but prominent deposits of C3 (now termed C3 glomerulopathy, C3G),^27^, ^28^ and a further subgroup of patients with electron dense deposits on glomerular and tubular basement membranes staining strongly for C3, the appropriately named dense deposit disease (DDD).^29^ Early studies showed no relation between plasma levels of C3 and renal disease activity^30^ and, in MPGN, hypocomplementemia persisted after bilateral nephrectomy.^31^ A higher proportion of patients with DDD were found to be positive for C3Nef than other forms of MPGN.^32^ C3 metabolic studies using radio‐iodinated C3 showed that reduced C3 synthesis appeared to be the likely cause of C3 hypocomplementemia,^1^, ^22^ though the interpretation of these experiments may need revising in the light of uncertainty about the half‐life of complement C3d; when combined C3 and C5 metabolism was studied, C5 metabolism was found to be normal in the small number of C3 Nef positive patients studied.^33^ These findings fitted the clinical state of these patients who showed no signs or symptoms of severe systemic complement activation. The histology which most resembles DDD is light chain nephropathy^34^ (with proteinaceous electron deposits along glomerular and tubular basement membranes), raising the possibility that DDD is somehow related to the abnormal turnover of complement proteins creating an amyloid‐like pathology. Though they usually stain for complement, the exact composition of the argyrophilic dense deposits is not known. Later, evidence that DDD was a consequence of uncontrolled complement activation came when Levy and her colleagues^35^ reported a family where FH deficiency was associated with glomerular disease with dense intramembranous deposits. FH deficiency was later found in a strain of pigs with MPGN, but the strain has now been lost.^36^

Detailed studies of nephritic factors (NeF) then showed that these autoantibodies are heterogenous; Nef that stabilized cell bound but not fluid phase C3 convertase,^37^ Nef that stabilized the classical pathway convertase,^38^, ^39^ Nef requiring Properdin,^40^ and Nef that activated the terminal pathway,^41^ the last associated with low plasma levels of C5, were all reported. In a study^42^ of a relatively large group of patients, C3Nefs were subdivided into two groups, one requiring Properdin (P) to stabilize the C3bBb convertase and the other not. The latter was predominantly found in DDD, whereas P‐dependent Nef occurred in GN with isolated C3 deposits and immune complex MPGN. In a case of Nef‐negative DDD, an autoantibody to FB (binding to an epitope in Bb) was found to produce complement deregulation.^43^ In a recent study of C3G and related disorders in children, a significant proportion had antibodies to individual complement components, FH, FB, and C3b.^44^

Hypocomplementemia is also a characteristic feature of acute post‐streptococcal nephritis (AGN) and was widely assumed to be a reflection of immune‐complex formation, with the majority of patients showing reduced concentrations of the early classical pathway components. However, in a small proportion of these patients, a C3 splitting factor active in Mg EDTA was found.^32^ Lately, antibodies to FB have been identified in a majority of patients with AGN.^45^ However, difficulties remain in correlating the forms of Nef with pathology, due to lack of standardization of the various assays for Nef, which are largely conducted in research laboratories^46^ Autoantibodies to FH, FB, FI, and C3b have also been found in atypical HUS, and presumably functionally mimic genetic deficiency of the control proteins.^15^

## 5 NEPHRITIC FACTORS AND IMMUNOCONGLUTININS

This extraordinary diversity of autoantibodies to complement epitopes presumably generated or revealed as a result of complement activation, suggests a low threshold for auto‐immunity and/or a high degree of antigenicity. Immunoconglutinins are usually IgM, but sometimes IgG, autoantibodies to fixed complement determinants (Lachmann 1967).^47^ Early work had shown them to be induced by injection of bacteria, presumably reacting to complement fixed on bacteria. This led Coombs and Coombs 1953^44^ to suggest that immunoconglutinins should be regarded as “physiogenic” autoantibodies because they have the potential of enhancing bacterial opsonization. Lachmann (1966),^48^ using a sensitive test for immunoconglutinins, found that no human sera tested entirely negative suggesting a degree of complement fixation was the normal state of affairs, an early premonition of the tickover hypothesis. High titers of immunoconglutinins were found in various rheumatic and autoimmune diseases, in patients with chronic infection, and in acute post‐streptococcal nephritis.^49^ Whether the seeming ease with which complement is auto‐antigenic might be due to lack of in vivo complement fixation and exposure of potentially antigenic epitopes in the sterile in utero environment or whether it is in some way related to the role of C3 in the afferent limb of immunity^50^ is an interesting conjecture.

## 6 MORE CASE REPORTS: CLUES FROM PARTIAL LIPODYSTROPHY (PLD)

Alper et al.^51^ in an early case of C3 hyper‐catabolism associated with susceptibility to infection, but no evidence of renal disease noted that the patient suffered from partial lipodystrophy. Serological studies revealed a “C3‐ase” and a complement profile similar to that of patients with Nef‐associated glomerulonephritis. PLD was long known to be associated with renal disease, but this did not develop in all patients.^52^, ^53^ This raised the possibility that C3Nef might be common to both conditions: typical complement profiles and C3Nef were then shortly identified in a group of patients with PLD who had no evidence of nephritis.^54^ Thus, it seemed that C3Nef predisposed to both PLD and nephritis, but neither outcome was inevitable. It also followed that C3Nef should be found in healthy subjects and in due course this was reported.^55^ A further development was the identification of a patient with PLD and low serum C3 with extensive retinal drusen, but no evidence of kidney disease.^56^ At about the same time, it was reported that patients with MPGN developed early retinal disease, with extensive drusen deposits resembling those found in AMD.^57^ This was the first and largely ignored clue for a role for complement in retinal pathology. Drusen were later reported in patients with DDD associated with FH mutations.^58^ In a recent paper, the same group conducted systematic examination of various patients with renal disease and suggested that drusen in young patients might be regarded as a clinical sign of systematic complement activation.^59^

In 1989, studies of adipocyte biology revealed first that adipsin was identical with complement factor D (FD),^60^ and that adipocytes were capable of synthesis of C3 and FB.^61^ This led Mathieson et al. to show that NeF was capable of causing complement‐dependent lysis of adipocytes in vitro,^62^ and, in turn, raised the question of the extent to which complement‐dependent pathology is influenced by local synthesis of complement proteins. Andrews et al., taking advantage of patients differing in C3 allotypes between donor and recipient renal transplants, showed that C3 was synthesized by donor glomerular and tubular epithelium cells.^63^ A feature of DDD is its very high recurrence and early onset in transplanted kidneys,^64^ in contrast to the situation in PLD described above, where long periods of complement activation may precede renal disease. It is known that ischemia‐perfusion stimulates renal complement synthesis,^65^ raising the possibility that this might be a trigger facilitating C3Nef‐induced recurrence of DDD. An interesting finding in a group of patients with recurrent DDD in transplanted kidneys reported by Droz et al.^66^ and Leibowitch et al.^67^ was the normalization of hypocomplentemia, and disappearance of C3Nef shortly after transplantation. This had also been reported earlier by Vallota et al.^31^ Rapid reduction in the circulating concentration of an autoantibody suggests effective immunosuppression or the availability of large amounts of autoantigen or both, and even more surprising was the early appearance of dense deposits which did not stain for C3. No satisfactory explanation has been provided for these findings.

## 7 COMPLEMENT KNOCKOUTS

The scene was set for a series of informative complement knockout studies in mice by Pickering et al.^68^, ^69^, ^70^ A notable experiment showed that FH‐deficient mice developed dense intramembranous glomerular deposits, but when the *FB* gene was also deleted, no disease developed. Also informative was that FI knockout protected FH‐deficient animals, showing the role of iC3b in renal pathology (presumably through its interaction with CR3 on neutrophils). This observation provided an explanation for the failure of FI deficiency to be associated with DDD in man; glomerulonephritis quickly followed infusion of normal FI‐containing plasma. C5‐deficient, but not C6‐deficient, mice were protected, and, in a related system where antibody to GBM nephritis was induced in FH knockout mice, mice had longer and persistent glomerular infiltration with neutrophils compared to control animals, and inhibition of C5 by a monoclonal antibody provided anti‐inflammatory protection.^71^ While these experiments indicate the importance of the inflammatory role of C5, they also demonstrate the pathogenic power of two‐hit immunopathology. However, it is important to record that in a different system where persistent complement activation was induced in mice by cobra venom factor (CVF), a powerful activator of the alternative pathway, no changes were detectable on electron microscopy after 3 months^72^ and mice transgenic for CVF were found to be healthy with a normal life‐span, despite systemic complement activation; detailed renal histology was not reported.^73^

There are now a substantial number of reports of FH deficiency in DDD.^74^ Even more subtle variations in local control of C3 amplification have been revealed by the findings of mutations in complement FH‐related (CFHR) proteins in a familial nephropathy first described in Cypriot families, where the resulting dimerization of the CFHR5 protein confers avidity for tissue bound complement fragments, increasing their capacity to compete with FH for ligand binding, thereby enhancing local complement activation.^75^, ^76^, ^77^

Thus, there is an impressive body of evidence to indicate that complement dysregulation of the amplification cycle is the major predisposing factor in this group of diseases. Against a background of gross deregulation of the C3bBb amplification cycle, otherwise minor inflammatory events such as transient immune complex deposition, or minor degrees of endotoxinemia, can induce persistent renal inflammation, maybe with the participation of locally synthesized complement proteins. It should also be considered that C3 depletion may play a role through failure of normal processing of immune complexes,^78^, ^79^, ^80^ and it is worth emphasizing that C3Nef is also associated with MPGN a typical immune complex nephropathy with Ig deposition in the kidney.^32^ Why broadly similar genetic or acquired dysfunction of the C3 amplification loop predisposes to the pathologies as distinctive as C3G or atypical HUS, or indeed may not be sufficient to cause either, is a tantalizing question. The molecular heterogeneity of the pathophysiology of these very rare renal diseases creates a challenge for therapeutic intervention unless a common pathogenic pathway such as C5 activation plays a dominant role, as was the case in Pickering's FH‐deficient mice that were protected by an anti‐C5 antibody.

## 8 COMPLEMENT IN THE GENOMIC ERA

These major disturbances of complement physiology causing rare renal diseases attracted only modest interest from the pharmaceutical industry. This changed dramatically with the publication of 3 papers in 2005 indicating that a single‐nucleotide polymorphism of FH was an important risk factor for AMD.^81^, ^82^, ^83^ The major risk factors of AMD are age, smoking, and genetic background. Genetic risk is particularly associated with polymorphisms in the complement FH *CFH* Y402H gene and the AMD susceptibility 2 ARMS2 A69S gene. AMD is characterized by drusen, complement‐containing deposits at the interface of the retinal pigment epithelial cells and Bruch's membrane, an anatomical arrangement that is similar to that in the renal glomerulus (the glomerular basement membrane which lies between glomerular capillary endothelial cells and the glomerular podocytes).

There followed an explosion in the discovery of complement allotypes and polymorphisms and their association with AMD,^84^, ^85^, ^86^ which led Harris, Morgan, and their colleagues to propose the concept of the complotype,^87^ a genetic description of the integrated effect of genetic variants in complement genes which influences risk for inflammatory disorders and infectious diseases. A pro‐inflammatory complotype, while offering an advantageous response to infection before adaptive immunity is fully developed, predisposes to complement‐mediated tissue injury from lifelong environmental inflammatory stimuli, in old age. The retina seems to be the most sensitive target, or where injury is most readily ascertained, and synergy between complement dysregulation and cigarette smoke, the major identified environmental risk factor for AMD, seems likely.^88^ Aged FH‐deficient mice developed retinal abnormalities, accelerated by exposure to cigarette smoke.^89^, ^90^ An interesting observation was that amyloid beta, a constituent of drusen, inhibited the ability of FI to cleave C3b to inactivated iC3b.^91^ The search for rare genetic variants conferring high risk of AMD disease also led to the identification of mutations in genes encoding FI.^92^, ^93^, ^94^, ^95^, ^96^ Kavanagh et al.^97^ reported mutations associated with significant reductions in plasma FI concentrations. These were the same mutations that had earlier been associated with atypical HUS, findings which reinforced ideas about the possibility of enriching plasma FI as a therapy for AMD (see later).

## 9 COMPLEMENT THERAPEUTICS

AMD is a major cause of blindness, and the commercial potential of anti‐complementary therapy was seized upon by the biotechnology and pharmaceutical communities. Complement was transformed from a niche subject into big business, with many of the large pharmaceutical companies establishing research programs, and with regular industry complement discovery conferences. A detailed discussion is beyond the scope of the review but I will briefly consider aspects that are related to the pathophysiology of the amplification loop and especially to Peter Lachmann's initiative in this area.

IN PNH, arrest of hemolysis provides an easily‐read indicator of the efficacy of anti‐complement therapies, but their development in renal and retinal diseases presents a formidable challenge. The renal diseases are rare, and, as we have seen, distinct molecular disorders of the system can produce similar pathologies. Further there is temporal heterogeneity brought about by the mix of immunopathology, inflammation, and post‐inflammatory scarring. AMD, by contrast, is common but is a slowly progressive condition of the elderly. Many decades of complement dysregulation occur before disease is manifest. The dry form of AMD, geographic atrophy, has presently no approved treatment. However, even in the much more extreme pathophysiology of the feedback cycle characteristic of DDD, it may take several decades for retinal disease to become apparent.

The C3 amplification loop is common to all pathways of complement activation and the major variable controlling the extent of complement activation. To dampen the cycle, the therapeutic options are obvious. The first is to develop inhibitors of its activators, C3, FB, Properdin, and FD. The second is to replace deficient inhibitors, to enrich such inhibitors, or to create new chemical entities with similar properties. Additionally, in the case of Nef‐driven disease, there is the option to deploy immunosuppressive anti‐B cell therapies and/or deplete autoantibodies by plasma exchange. (In the last case, the lack of correlation between complement serology and disease activity is a major concern.) An alternative approach is to inhibit the inflammatory complement molecules C3a and C5a or block the MAC. Over several decades, all these approaches have been or are being explored in renal and/or retinal complement pathologies.

To date, only four anti‐complement therapies have been approved: C5 inhibition with eculizumab, of great benefit in PNH and aHUS, more recently approved for the treatment of myasthenia gravis (MG) and neuromyelitis optica (the eculizumab follow‐up antibody, ravulizumab, with a prolonged half‐life, has also been approved for the treatment of PNH, aHUS, and MG); avacopan, a specific inhibitor of C5a approved for the treatment of ANCA vasculitis^98^; pegcetacoplan, an inhibitor of C3 activation in PNH; and sutimlimab (a C1s inhibitor) in cold agglutinin disease. A controlled trial of avacopan in C3 Glomerulopathy is current being reviewed (NCT03301467). In this trial, it would be desirable to stratify C3G patients according to involvement of C5, or at least according to evidence of the neutrophil‐dependent renal inflammation which C5a would be expected to induce. Potent inhibitors of C3, FB, and FD are currently in clinical trials for C3G and/or AMD, some with encouraging preliminary findings, see ClinicalTrials.Gov, and.^99^, ^100^ In the case of AMD, local inhibition of complement activation has obvious attractions and the use of intravitreal therapy for wet AMD with anti VEGF well established. The C3 inhibitor from Apellis, pegcetacoplan, administered by monthly intravitreal injections, recently completed a phase 3 trial for dry AMD (NCT04770545).

In 1975, Lachmann and Halbwachs^101^ showed that enrichment of FI by modest amounts (approximately 25 percent) suppressed activation of the alternative pathway by powerful activators such as inulin or LPS, and suggested that infusion of FI would be a rational and safe therapy in patients. After failing to persuade various biotech and pharma companies to produce therapeutic quantities of FI, Lachmann in 2014 helped found a company, Gyroscope Therapeutics, a specialist gene therapy company. Gyroscope has developed a system whereby a *single* injection of a vector encoding FI is injected into the subretinal space in patients with AMD. Starting with the subgroup with low FI levels referred to above, the first patient in a phase 1 trial was treated in 2019,^102^ more than 4 decades after the 1975 paper was first published. Phase 2 studies are now in progress (NCT04437368, NCT04566445). Gyroscope was purchased by Novartis early in 2022 just over a year after Peter Lachmann's death in December 2020.

## CONFLICT OF INTEREST

Chair of the Scientific Advisory Board of Gyroscope Therapeutics.

## Data Availability

This review contains no unpublished data.
